# Peroneal Artery Danger Zone With Syndesmotic Screw Fixation: A Computed Tomography Angiography Study

**DOI:** 10.1177/10711007261422699

**Published:** 2026-03-17

**Authors:** Garrett K. Berger, Avinaash Korrapati, Aaron Tran, Zachary Brumm, Sean Thomas, Kevin Y. Zhu, William T. Kent

**Affiliations:** 1 Department of Orthopaedic Surgery, University of California–San Diego, San Diego, CA, USA; 2 School of Medicine, University of California–San Francisco, San Francisco, CA, USA

**Keywords:** syndesmosis, peroneal artery, syndesmotic screw, unstable ankle, vascular injury

## Abstract

**Background:**

Additional trans-syndesmotic screws (TSS) are used in “fibula pro-tibia” approach for unstable ankle fractures applied to high-risk patients (diabetes, smoking, osteoporosis, obesity). These patients are at higher risk of complications. The peroneal artery (PA) may be at risk with additional TSS fixation. Iatrogenic vascular compromise may explain these postoperative complications. Therefore, this study investigates the range in which the PA and its deep perforating branch (dPA) are at risk with TSS fixation and identifies safe zones to avoid iatrogenic injury in this vulnerable population.

**Methods:**

A retrospective analysis of lower extremity computed tomography angiograms (CTAs) was performed (2021-2022) in specified patients with comorbidities who might benefit from multiple syndesmotic screw fixation. CTAs were reformatted in the syndesmotic plane, and the PA was deemed at risk if a templated 3.5-mm syndesmotic screw intersected its course. Measurements were taken from both the tibial plafond and fibular tip and included the level where the PA and dPA branch entered and exited this danger zone.

**Results:**

Ninety-eight CTAs (196 limbs) were analyzed. Seventy-two patients were age ≥ 65 (mean 75, SD 8), 52 had diabetes, 16 were active nicotine users, and 16 had BMI ≥35. The PA was at risk in 98.5% (n = 195) limbs. The danger zone began 7.5 cm (SD 1.5 cm) proximal to the tibial plafond and 10 cm (SD 1.5 cm) proximal to the fibular tip. The dPA branch perforated the syndesmotic plane at 3.6 cm (SD 0.8 cm) proximal from the plafond and 6 cm (SD 0.9 cm) from the fibular tip. Finally, the PA and dPA were out of the danger zone at 2.5 cm (SD 0.4 cm) proximal from the tibial plafond and 5 cm (SD 0.6 cm) from the fibular tip. No difference was found between inclusion subgroups nor between individual patients’ contralateral legs.

**Conclusion:**

PA and dPA are at risk with TSS, notably in the distal fifth of the limb, ending ~2.5 cm proximal to the plafond. Knowledge of this zone aids in planning for TSS fixation, especially for high-risk patients.

**Level of Evidence:** Level IV, case series.

## Introduction

At a rate of 4.22 per 10 000 person-years, ankle fractures are among the most common orthopaedic injuries in the United States.^1^ Often, these injuries are accompanied by an injury to the distal tibiofibular syndesmosis.^2,3^ Diastasis of the syndesmosis, if left untreated, can lead to decreases in talocrural contact and subsequent progression of post-traumatic ankle osteoarthritis.^4^ Therefore, if the syndesmosis is found to be unstable, various surgical fixation techniques have been used, including transsyndesmotic screws (TSS), suspensory fixation constructs, and hybrid aperture fixation devices.

Classically, with single TSS fixation, the start point is approximately 1-2 cm proximal to the tibial plafond.^5,6^ However, patients with poor bone quality and/or neuropathy (diabetes patients, obese patients, smokers, elderly) are at higher risk failure of fixation.^7^ Commonly, the dogmatic principle of ‘double the fixation’ is applied to these patients. As such, they often are treated with multiple syndesmotic screws for increased stability.^8-10^ There are numerous examples in recent literature describing ‘fibula pro-tibia’ fixation for unstable ankle injuries, wherein several syndesmosis screws are placed at the distal third junction of the fibula.^11,12^ This technique is also advocated for in the revision nonunion setting, one in which skin viability is often of concern.^13^ These same high-risk patients are also at higher risk of postoperative complications, such as wound healing issues and infection.^8-10^ Many studies have found a compromised and decreased vascular supply to contribute directly to these higher rates of complications.^14,15-17^ Despite the increasing use of this technique, there remains a scarcity of literature available regarding safe placement for more proximal TSS.^18^ Specifically, to avoid damaging the angiosome fed by the peroneal artery supplying the posterolateral lower leg, ankle and heel.^19^ As arterial insufficiency in these patients can contribute toward higher risk of complications and fixation failure, further insults to the vasculature, potentially from TSS, could further increase the risk of postoperative complications especially in the peroneal artery angiosome.^20,21^

**Figure 1.**
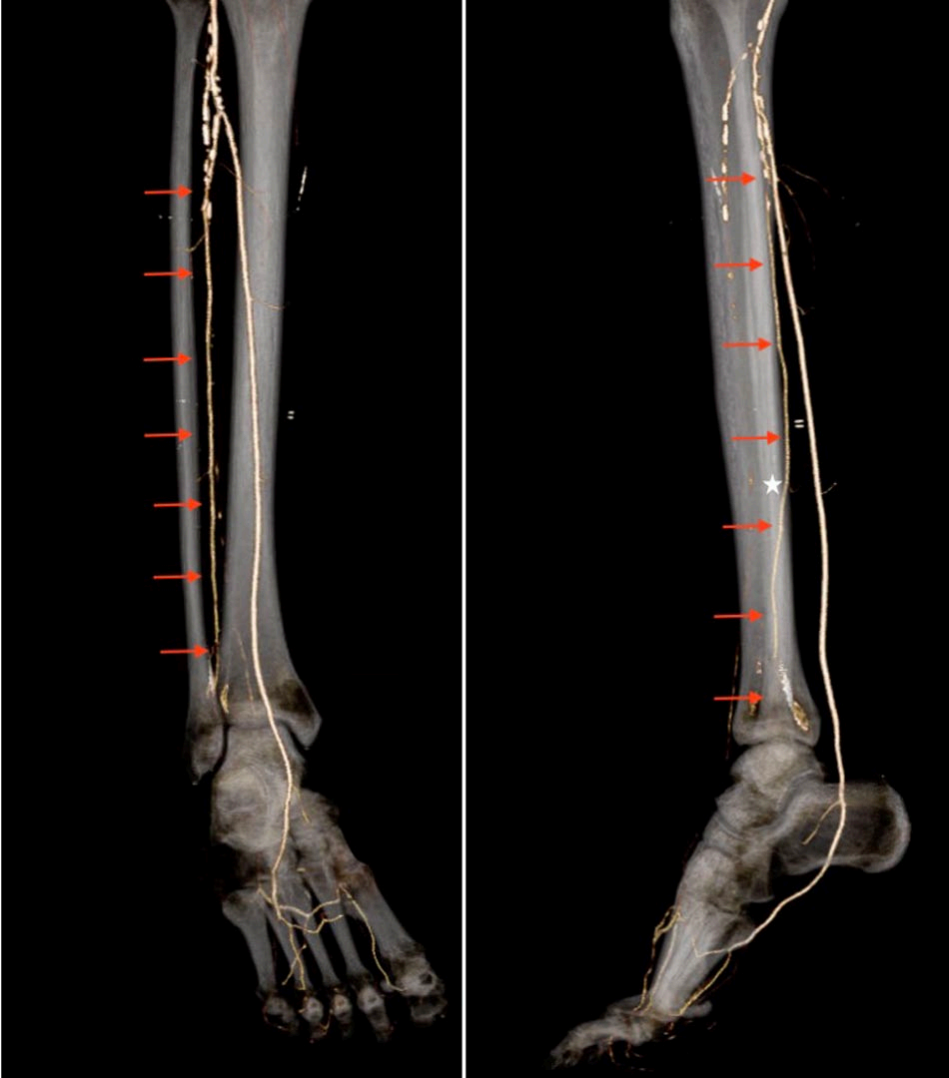
Three-dimensional reconstructions of the computed tomography angiogram with the peroneal artery highlighted by red arrows. Notice the point at which is crosses anteriorly into the syndesmotic plane (white star). [See online article for color figure.]

The peroneal artery (PA) and its branches, specifically the deep perforating branch (dPA), parallel the posterior border of the fibula and cross anteriorly as they travel distally to anastomose with the anterior tibial artery and provide collateral circulation to the hindfoot and ankle (Figure 1).^22^ Particularly in the trauma setting, blood supply to the skin and soft tissues is critical for wound healing. There have been limited reports of arterial injury with TSS fixation, although the downstream effects of these injuries have not been evaluated.^23,24^ To date, no studies have evaluated the range in which the peroneal artery (PA) and its deep perforating branch (dPA) are at risk with TSS fixation. Therefore, the purpose of this study is to examine where TSS fixation may compromise the PA and dPA and determine if safe zones exist to achieve rigid, stable fixation in patients requiring it.

## Methods

**Figure 2.**
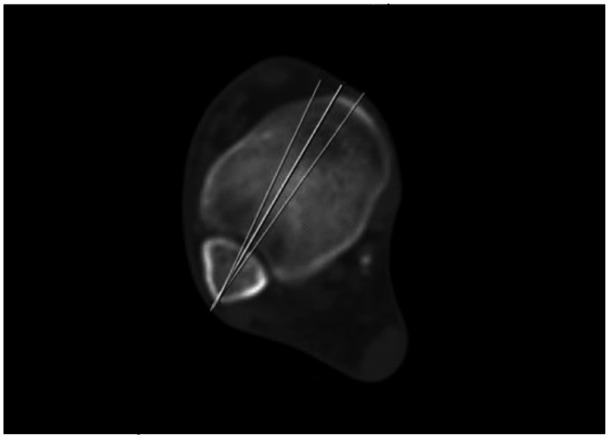
On the bone window, the syndesmotic plane is established, and a 5-degree margin for error is accounted for anteriorly and posteriorly.

**Figure 3.**
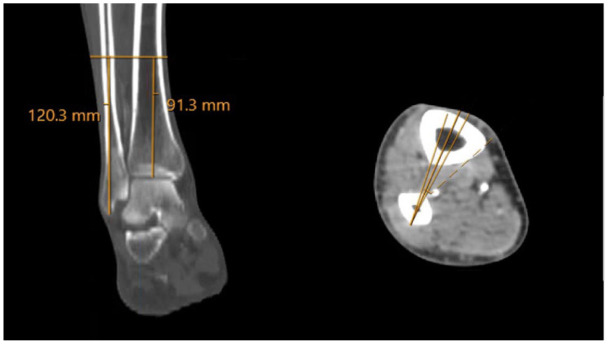
The syndesmotic plane is established proximally, with the aforementioned 5-degree margin for error. Perpendicular to this, a 3.5-mm line is drawn to represent the diameter of a 3.5-mm transsyndesmotic screw. On the soft tissue window, the point at which the peroneal artery intersects the screw is marked on the coronal plane. The distance from the tibial plafond and fibular tip to this cut is measured and deemed the start point of the danger zone.

**Figure 4.**
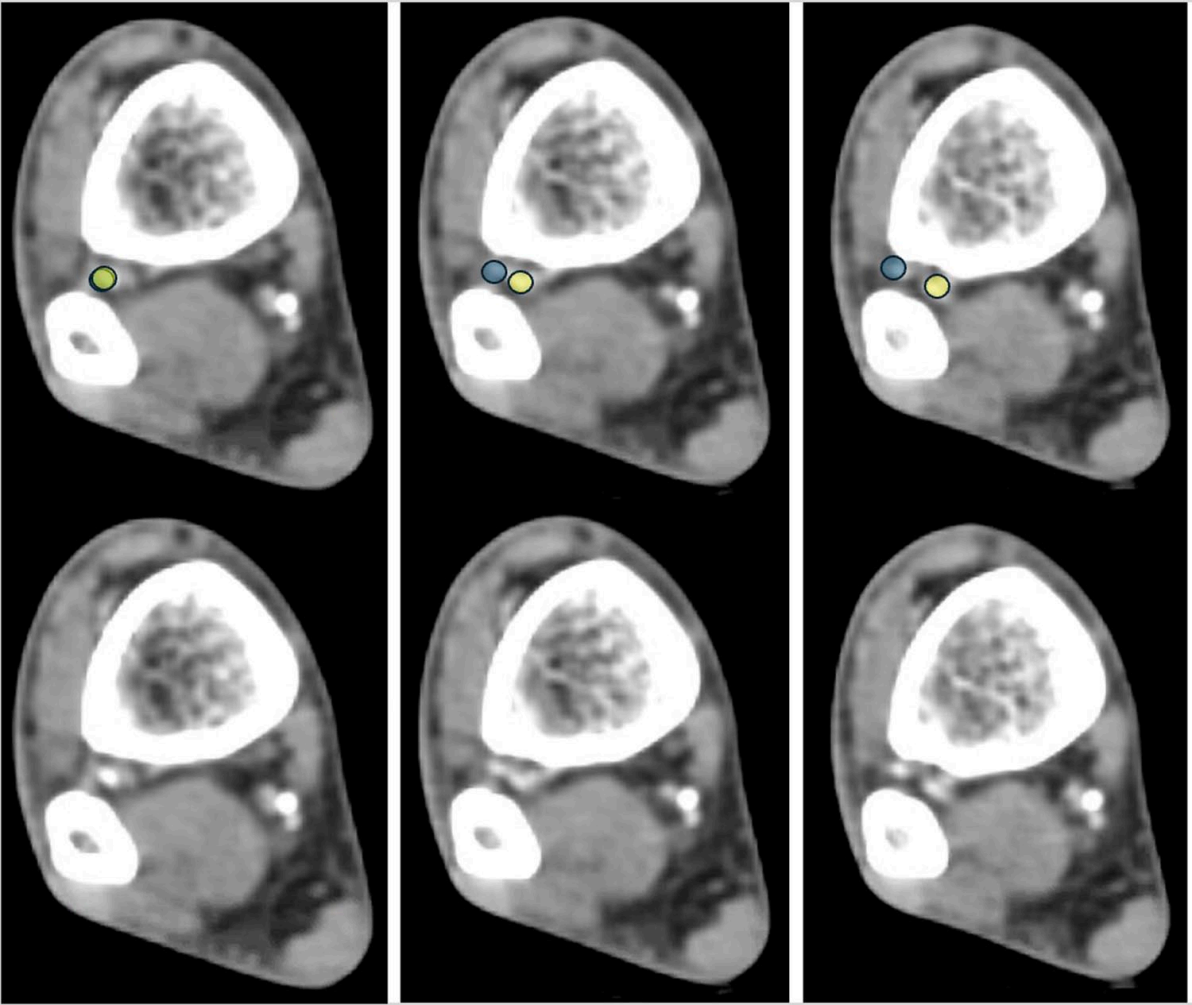
Next, on the soft tissue window, the level at which the deep perforating branch of the peroneal artery (dPA) branches off is measured. Here, 3 representative cuts (3 mm apart for illustrative purposes) are shown with and without the yellow (peroneal artery) and blue (deep perforating branch) overlays. [See online article for color figure.]

**Figure 5.**
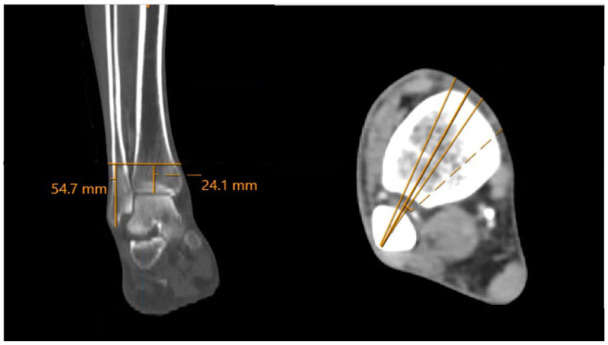
Next, the process is repeated to measure the end of the danger zone, as both the residual peroneal artery (anterior) and the deep perforating branch (posterior) exit the danger zone.

A retrospective analysis of uninjured lower-extremity computed tomography angiograms (CTAs) performed between 2021 and 2022 was performed at an academic level 1 trauma center. Inclusion criteria specified particular patient demographics and comorbidities who would be indicated from multiple syndesmotic screws including age ≥65 years, diagnosed with diabetes (A1c ≥ 6.5%), active nicotine use, and obese patients (BMI ≥ 35). Exclusion criteria specified patients with arterial disease impairing the ability to visualize the PA or dPA bilaterally, an acute fracture, history of fracture at the ankle/distal tibia, or malunion (as this would potentially distort normal anatomy), lower extremity amputation, and abnormal anatomy secondary to arterial bypass procedure. CTAs were reformatted to be in the syndesmotic plane (20 degrees posterior to the coronal plane, parallel to the tibial plafond), and the PA was deemed to be at risk if a templated 3.5-mm syndesmotic screw intersected its course within a 5-degree angular margin for error anteriorly and posteriorly.^25-27^ Measurements were taken from both the tibial plafond and fibular tip to the level at which the PA entered and exited this danger zone as well as the level of the dPA branch. These measurements are depicted in Figures 2 to 5. Leg lengths were also measured from the tibial plateau to the tibial plafond, bilaterally. Danger zones were further characterized as a percentage of leg length, so as to account for height differences in clinical application. Statistical differences were analyzed with *t* test and analysis of variance, through SPSS (version 28).

### Ethics Statement

This study was reviewed and determined to be exempt from institutional review board (IRB no. 191168) oversight because of its retrospective design and use of deidentified data, in accordance with institutional policies, federal regulations governing human subjects research, and in accordance with ethical standards in the 1964 Declaration of Helsinki.

## Results

**Figure 6.**
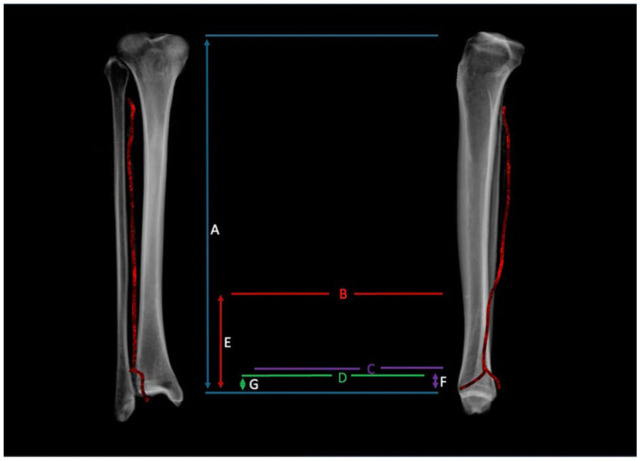
(A) Limb length as measured from tibial plateau to plafond. (B) The entry point at which the PA courses anteriorly and enters the “danger zone.” (C) The branch point of the dPA. (D) The point at which the danger zone ends. (E) The distance from tibial plafond to the start of the danger zone (average 7.5 ± 1.5 cm). (F) The distance from the tibial plafond to the dPA (3.6 ± 0.8 cm). (G) The distance from the tibial plafond to the start of the danger zone (average 2.5 ± 0.4 cm). The ratio of E/A was 20% (SD 4%) in our cohort.

Ninety-eight CTAs (196 limbs) were analyzed. Seventy-two patients were 65 years of age or older (mean 75 years, SD 8), 52 had diabetes (mean A1c 7.3, SD 1.4), 16 were active nicotine users, and 16 had BMI ≥35 (mean 39 ± 5) (Table 1). Average limb length was 36.7 cm (range 30.2-42.5 cm, SD 2.7 cm) with an average limb length discrepancy of 2 mm (range 0-12 mm, SD 2 mm). The PA was at risk in 98.5% (n = 195) of limbs. On average, the danger zone began 7.5 cm (SD 1.5 cm) proximal to the tibial plafond and 10 cm (SD 1.5 cm) proximal to the fibular tip. Relative to limb length, this correlated to 20% of the limb length proximal from the plafond (SD 4%) (Figure 6). The dPA branch perforated the syndesmotic plane at 3.6 cm proximal from the plafond (SD 0.8 cm) and 6 cm (SD 0.9 cm) from the fibular tip. Finally, the PA and dPA exited the danger zone at 2.5 cm (SD 0.4 cm) proximal from the tibial plafond and 5.0 cm (SD 0.6 cm) from the fibular tip. No difference was found between inclusion subgroups or between individual patients’ contralateral legs. These data are further characterized in Table 2.

**Table 1.** General Demographics.

Demographic Variables		Percent
Total	98	100
Age ≥ 65 y		
Never smoker	72	73
Current smoker	26	27
Smoking history		
Nonsmoker	86	88
Smoker	12	12
Diabetes with A1c ≥6.5		
No	69	70
Yes	29	30
BMI ≥35		
No	82	84
Yes	16	16

**Table 2.** Danger Zone Measurements.

	Distance From Tibial Plafond			Distance From Fibular Tip		
	Mean (cm)	Range (cm)	SD (cm)	Mean (cm)	Range (cm)	SD (cm)
DZS DZS as a % of LL	7.5 20.2	4.5-11 12-31	1.5 4	10.1	6.1-13	0.9
dPA branch	3.6	2.3-8.6	0.9	6.0	3.3-11	1.0
DZE	2.5	1.5-4.7	0.5	4.9	2.8-7.3	0.6

Abbreviations: dPA, deep perforating branch of the peroneal artery; DZE, danger zone end; DZS, danger zone start; LL, limb length.

## Discussion

This is the first study describing the danger zone for TSS fixation with respect to the peroneal artery (PA) and its deep perforating branch (dPA). Our findings indicate that the danger zone begins approximately 7.5 cm proximal to the tibial plafond (~20% of tibial length) and ends approximately 2.5 cm proximal to the tibial plafond. The PA and dPA are at risk in virtually our entire (99.5%) study population.

Several anatomic studies have evaluated various aspects of the PA and dPA, all of which were cadaveric studies. Attinger et al^19^ used colored methyl methacrylate in 50 cadaver dissections to reconstruct patient’s vascular anatomy. For the peroneal artery angiosome, they found its supply to the skin extends from the midline posterior calf to the anterior edge of the lateral compartment and continues inferiorly to the lateral and inferior heel and anterolateral ankle. Penera et al^3^ focused on evaluation of the dPA. They found that the dPA perforated the interosseous membrane at approximately 3.4 cm proximal to the tibial plafond, consistent with our finding of 3.6 cm. However, the major conclusion from this study is that TSS proximal to 4 cm from the plafond is safe and will not damage the dPA. Although true with respect to the dPA directly, based on our research, transsyndesmotic screws proximal to this are likely to damage the PA itself, and therefore impact the dPA indirectly. This may compromise the blood supply to the syndesmotic ligaments and the skin and soft tissues of the lateral ankle and hindfoot. Exemplifying this is the cadaveric study by McKeon et al,^28^ who found that the dPA perforated the interosseous membrane at approximately 3 cm proximal to the tibial plafond, and that it was the primary blood supply to the anterolateral ligaments in 63% of specimens. Lidder et al^29^ performed a cadaveric study via a posterolateral approach, evaluating the bifurcation of the PA. They found that the bifurcation occurred on average 8.3 cm proximal to the tibial plafond and that the dPA perforates the interosseous membrane at an average of 6.4 cm proximal to the tibial plafond, more proximal than the findings from our study or the aforementioned Penera et al^3^ study. Although there are multiple angiosomes that provide perfusion to the foot and ankle, this skin and soft tissue is often tenuous in the trauma setting and can lead to catastrophic complications, if compromised.^30^ Similarly, despite collateral flow, Attinger et al^19^ found only certain arteries would supply the critical blood flow to specific areas in the foot and recommended using doppler assessment to determine dominant supply prior to revascularization due to this variability. This concept is particularly important for diabetes patients or those with peripheral vascular disease, as the primary critical blood can easily be compromised. In a similar fashion, 15% of arterial bypasses fail to revascularize the foot despite the bypass remaining patent; this is due to this concept of the bypass failing to revascularize the dominant angiosome to the specific area of the foot.^21^ Despite collateral circulation and contributions of the anterior tibial and posterior tibial arteries, studies show that healing in the lateral ankle and hindfoot continues to be challenging even after revascularization. This concept is especially important in patients with a dominant PA, or peroneal magnus artery, which accounts for 5% of the total patient population.^22^ Patients with this physiologic variance have a larger than normal PA while having smaller than normal anterior and posterior tibial arteries. In those who have comorbidities causing microvascular disease, where the anterior and posterior tibial arteries would be more likely affected, iatrogenic damage to the dominant peroneal magnus artery could be devastating as these patients have a higher incidence of surgical complications (ie, foot/limb ischemia) already.^22^ Therefore, every effort to preserve blood supply to this area should be considered in the traumatized foot and ankle, especially in patients with already compromised vasculature from their comorbidities. To date, there are no studies evaluating postoperative skin and soft tissue complications following more proximal syndesmotic fixation in the form of fibula pro-tibia fixation.

Although evidence does support that the PA may be at risk in zones where multiple syndesmotic screws may be used, the question remains as to alternatives to multiple screw use in this patient population. Dissection to identify and protect the PA in these zones is truly only feasible with a posterolateral approach (often used to approach fractures both requiring fixation of the fibula and buttress of the posterior malleolus). More commonly a lateral approach to the distal fibula is performed when placing TSS. With a lateral approach, the PA is not visualized, and placement of TSS need to be at the distal 2.5 cm of the fibula (to the plafond) or proximal to 7.5 cm to avoid the zone of potential injury with screw placement. It is possible that in this high-risk patient population, preoperative CTAs are available in the workup and treatment of vasculopathies associated with several comorbidities. In these instances, review and preoperative TSS placement can be conducted to best avoid injury to the PA.

This study is limited by several factors, one of which is the inherent bias of a retrospective study. The study population is limited to only patients who obtained a CTA, which may be a selection bias, although our cohort was relatively consistent across the board with low variation. Patients who were excluded based on having undergone a vascular bypass procedure also present a limitation, as these patients fall within the at-risk demographic. However, based on study design, we were not able to properly evaluate these patients, and focused rather on defining normal anatomy. Further, there are no prospective studies demonstrating compromising these arterial structures with drill bit or screw placement leads to poorer clinical outcomes. Future studies are needed to determine the risk of wound complications and healing rates in high-risk ankle fracture patients receiving TSS within this danger zone.

## Conclusion

The peroneal artery and deep perforating branch of the peroneal artery are at risk with transsyndesmotic screw fixation, most notably in the distal fifth of the limb from 7.5 to 2.5 cm proximal to the plafond. Knowledge of this zone can aid in planning for those high-risk patients (ie, diabetes, smoking, osteoporosis, obesity) who might benefit from multiple syndesmotic screws and to avoid potential complications (ie, loss of fixation and Charcot arthropathy) from vascular compromise and poor angiosome healing.

## Supplemental Material

sj-pdf-1-fai-10.1177_10711007261422699 – Supplemental material for Peroneal Artery Danger Zone With Syndesmotic Screw Fixation: A Computed Tomography Angiography Study Supplemental material, sj-pdf-1-fai-10.1177_10711007261422699 for Peroneal Artery Danger Zone With Syndesmotic Screw Fixation: A Computed Tomography Angiography Study by Garrett K. Berger, Avinaash Korrapati, Aaron Tran, Zachary Brumm, Sean Thomas, Kevin Y. Zhu and William T. Kent in Foot & Ankle International
